# Organ Donation for Research Biobanking Among Historically Marginalized Racial and Ethnic Groups: A Systematic Review

**DOI:** 10.1001/jamanetworkopen.2025.12133

**Published:** 2025-05-27

**Authors:** Camilo Toro, Oseiwe B. Eromosele, David B. Flynn, Andrew A. Wilson, Darrell N. Kotton, Timothy M. Hughes, Jesse D. Moreira-Bouchard, Wendy S. Post, Alain G. Bertoni, Emelia J. Benjamin, Deepa M. Gopal, Jessica L. Fetterman

**Affiliations:** 1 Evans Department of Medicine and Whitaker Cardiovascular Institute, Boston University Chobanian and Avedisian School of Medicine, Boston, Massachusetts; 2 Medical Sciences and Education, Boston University Chobanian and Avedisian School of Medicine, Boston, Massachusetts; 3 Center for Regenerative Medicine of Boston University and Boston Medical Center, Boston, Massachusetts; 4 The Pulmonary Center, Department of Medicine, Boston University Chobanian and Avedisian School of Medicine, Boston, Massachusetts; 5 Department of Gerontology and Geriatric Medicine, Wake Forest University School of Medicine, Winston-Salem, North Carolina; 6 Division of Cardiology, Department of Medicine, Johns Hopkins University, Baltimore, Maryland; 7 Department of Epidemiology, Bloomberg School of Public Health, Johns Hopkins University, Baltimore, Maryland; 8 Section of Cardiovascular Medicine, Department of Medicine, Boston Medical Center, Boston University Chobanian and Avedisian School of Medicine, Boston, Massachusetts; 9 Department of Epidemiology, Boston University School of Public Health, Boston, Massachusetts; 10 Department of Public Health Sciences, Wake Forest University School of Medicine, Winston-Salem, North Carolina

## Abstract

**Importance:**

Research biobanks of human cells and tissues, particularly tissues accessible only after death, are crucial for advancing the understanding of human pathophysiological function. Research biobanks are bereft of tissues from individuals of diverse races and ethnicities, thus limiting the generalizability of biobank findings.

**Objective:**

To evaluate the barriers and facilitators to participation in postmortem brain donation for research among individuals from historically marginalized races and ethnicities.

**Evidence Review:**

The published literature in PubMed, Embase, Web of Science, and PsycINFO databases was searched from 1973 to January 1, 2024. Studies that were written in English that involved adult participants (aged ≥18 years) and explored attitudes, perceptions, and beliefs on solid organ donation were included. Studies in which organ donation was for transplant and not for research, studies examining pediatric organ donation, narratives or perspectives, and studies that did not examine attitudes or beliefs toward organ donation were excluded. Two authors independently coded and performed a thematic analysis of eligible studies.

**Findings:**

Eighteen studies met the inclusion criteria, which involved 12 124 participants across multiple self-reported races and ethnicities (eg, Black or African American, Chinese, Hispanic or Latiné, and White). Most studies (16 [89%]) evaluated perceptions, attitudes, and beliefs among Black or African American and Hispanic or Latiné individuals toward postmortem brain donation. Five themes that informed the decision to donate were identified: information and misconceptions about the organ donation process (16 studies [89%]), mistrust of the research and medical communities (9 studies [50%]), family involvement (9 studies [50%]), religious and cultural beliefs (9 studies [50%]), and altruism (7 studies [39%]). Mistrust and religious and cultural beliefs were largely barriers to considering postmortem organ donation for research. Misconceptions were common, particularly regarding the donation process and purpose of biospecimens for research. Family involvement was both a barrier and a facilitator. Altruism, particularly understanding that organ donation would benefit the participants’ family, future generations, and community, was a facilitator.

**Conclusions and Relevance:**

This systematic review suggests that sharing culturally sensitive information about the organ donation process, engagement of participants and family members in shared decision-making, and addressing barriers and facilitators to donor recruitment practices may increase participation in research biobanks of individuals from underrepresented racial and ethnic groups.

## Key Points

**Question:**

What are the key barriers and facilitators to postmortem brain donation for biobanking among members of historically marginalized racial and ethnic groups?

**Findings:**

In this systematic review of 18 studies with 12 124 participants, 16 evaluated attitudes and beliefs about postmortem organ donation for research among Black or African American and Hispanic or Latiné participants. Thematic analysis of the studies identified 5 key themes affecting the decision to donate: information and misconceptions, mistrust, family involvement, religious and cultural beliefs, and altruism.

**Meaning:**

The findings suggest that sharing information about the process of organ donation, engaging participants and next of kin, and addressing barriers and facilitators to donation may be effective in increasing the diversity of translational research participants.

## Teaser

This systematic review describes the perceptions of, participation in, and concerns regarding postmortem organ donation for research purposes among racial and ethnic minority individuals.

## Introduction

Since the 2001 publication of the human genome, systems biology approaches have grown exponentially with the advent of high-throughput technologies and large-scale bioinformatic analysis, which can be applied to human tissues.^1^ Peripheral blood is readily accessible and has provided insights into many pathologic states; however, the genomic signatures underlying health and disease states may be tissue specific, justifying the need for tissue- and disease-specific biobanks.

In the setting of cancer, diseased and healthy tissues from the same patient are often collected, facilitating research into the underlying biological process of many cancers, which have driven advances in precision medicine. The Cancer Genome Atlas, comprising tissues from more than 11 000 patients with clinical data and outcomes, has led to discoveries connecting genetic aberrations with cellular dysfunction. These findings have directly translated into improved classification and prognostication as well as novel therapeutic targets.^2,3,4,5^ Due to clinical inaccessibility, the collection of most human tissues and organs affected by common diseases necessitates postmortem tissue procurement.

Postmortem procurement of organs allows access to tissues from individuals without disease, a key limitation of tissue collection from clinical procedures, and access to tissues not accessible through clinical procedures. Organs may also be acquired at the time of transplant; however, such organs are typically from individuals with end-stage organ dysfunction requiring transplant or from donors who have been declined for transplant due to other comorbidities. Postmortem organ donation for research is often solicited from living prospective participants both for ethical reasons and to expedite the procurement and preservation process to minimize molecular degradation.^6,7^ Postmortem donation differs from tissue sample collection during clinical procedures as consent must ultimately be provided by the next of kin. Discussions regarding postmortem donation with prospective donors and next of kin require substantial sensitivity, considering both the subject matter and the timing of such conversations (close to death). Hence, best practices for facilitating such conversations are essential for effective recruitment of donors for postmortem organ biobanks for research.

People from marginalized racial and ethnic backgrounds are underrepresented in studies across the entire spectrum of research (eFigure in Supplement 1), limiting the generalizability of results.^8,9,10,11^ To build organ and tissue biobanks that better reflect the diversity of the population, we aimed to evaluate the barriers and facilitators to participation in postmortem organ donation for research among individuals from historically and intentionally marginalized racial and ethnic groups. Enhancing recruitment of individuals from marginalized racial and ethnic groups for postmortem organ donation will be essential for advancing the understanding of many chronic and prevalent diseases to improve classification and prognostication and to identify new therapeutic targets that benefit all people.

## Methods

### Literature Search

We conducted a systematic review of the published literature on the perceptions, beliefs, and attitudes of racially and ethnically marginalized populations about postmortem organ donation for research. On January 1, 2024, we searched PubMed, Embase, Web of Science, and PsycINFO with a medical librarian (D.B.F.). MeSH terms and text words for different racial and ethnic groups were combined with terms related to organ donation to identify relevant publications (Table 1). The search period was from 1973 to January 1, 2024. We followed the Preferred Reporting Items for Systematic Reviews and Meta-Analyses (PRISMA) reporting guideline.^12,13^

**Table 1.** Databases and MeSH Terms for Literature Search^a^

Concept	MeSH terms
Organ donation	((“Tissue and Organ Procurement” [MeSH] OR “Organ Donation” OR “Organ Donations”)
Historically marginalized racial and ethnic populations	(((“Ethnicity”[MeSH] OR “Ethnic Groups” OR “Ethnic Group” OR “Nationality” OR “Nationalities”) OR (“Black or African American”[MeSH] OR “Black Americans” OR “American, Black” OR “Black American” OR “Blacks” OR “Negroes” OR “Negro” OR “African Americans” OR “African American” OR “American, African” OR “Afro-American” OR “Afro American” OR “Afro-Americans” OR “Afro Americans” OR “African-Americans” OR “African-American”)) OR (“Hispanic or Latino”[MeSH] OR “Hispanic or Latinos” OR “Latinos” OR “Latino” OR “Latinx” OR “Hispanics” OR “Cuban Americans” OR “Cuban American” OR “Hispanic Americans” OR “American, Hispanic” OR “Hispanic American” OR “Spanish Americans” OR “Spanish American” OR “Puerto Ricans” OR “Puerto Rican” OR “Latinas” OR “Latina” OR “Latin Americans, US” OR “American, US Latin” OR “Latin American, US” OR “US Latin American” OR “US Latin Americans”)))
Solid organ donation	((((((((“Heart”[MeSH] OR Heart OR Hearts) OR (“Liver”[MeSH] OR Liver OR Livers)) OR (“Kidney”[MeSH] OR Kidney OR Kidneys)) OR (“Lung”[MeSH] OR Lung OR Lungs)) OR (“Spleen”[MeSH] OR Spleen OR Spleens)) OR (“Pancreas”[MeSH] OR Pancreas)) OR (“Brain”[MeSH] OR Brain OR Brains)) OR (“Intestines”[MeSH] OR Intestines OR Intestine))

Abbreviation: MeSH, Medical Subject Headings.

^a^ PubMed, Embase, Web of Science, and PsycInfor were searched.

### Inclusion and Exclusion Criteria

We included original published works written in English that involved adult participants (aged ≥18 years) and explored attitudes, perceptions, and beliefs on solid organ donation. We excluded studies without available full text; studies that examined organ donation for transplant and not for research purposes; studies with pediatric participants (aged <18 years); and reviews, editorials, or descriptive articles.

### Validity Assessment

Four of us (C.T., O.B.E., D.M.G., J.L.F.) screened the titles and abstracts for eligibility. Two pairs of coders (C.T., O.B.E., D.M.G., J.L.F.) evaluated the eligibility of studies independently. First, we excluded duplicates and studies without available full text (Figure). Second, studies that were not relevant were excluded in the following order: organ donation for nonresearch purposes, organ donation in children, narratives or perspectives, and no examination of attitudes and beliefs about organ donation. Third, when studies met the eligibility criteria, we obtained the full-text articles.

**Figure.**
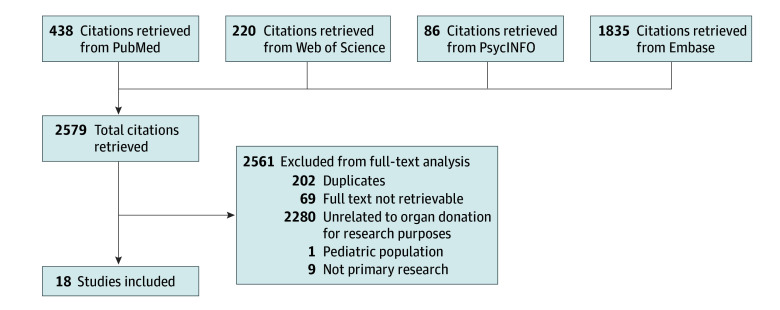
Flow Diagram of Study Selection PubMed, Embase, Web of Science, and PsycINFO were searched to identify relevant publications, using Medical Subject Headings terms and text words for different racial and ethnic groups combined with terms related to organ donation. Titles and abstracts were screened for eligibility, and eligibility of studies was independently evaluated. Full-text articles were obtained for studies that met eligibility criteria.

### Data Analysis

After all eligibility criteria were met, 2 of us (C.T., O.B.E.) independently performed a quantitative and thematic synthesis of each study. The quantitative assessment included extracting participants’ demographic information and consent rates by race and ethnicity, if reported. We used the 6-step thematic analysis described by Braun and Clarke^14^ to identify, analyze, and report patterns within the included studies. First, we familiarized ourselves with the studies through repeated reading and note-taking. Second, we generated initial codes systematically across the dataset using an inductive approach. Third, we grouped related codes into broader themes, refining them based on their relevance to barriers and facilitators. Fourth, we reviewed and refined these themes to ensure coherence and distinctiveness. Fifth, we clearly defined and named themes to encapsulate their central concepts. Sixth, we synthesized findings into a narrative format, illustrating key themes with representative examples from the included studies.

To enhance the trustworthiness of our analysis, 4 of us (C.T., O.B.E., D.M.G., J.L.F.) reviewed the results. Any disagreement was resolved by consensus.

## Results

The search yielded 2579 citations, with 2561 being ineligible after full-text review. Eighteen studies^15,16,17,18,19,20,21,22,23,24,25,26,27,28,29,30,31,32^ met the inclusion criteria (Figure). Not being related to organ donation for research (ie, donation for transplant) was the most frequent reason for article exclusion.

The study characteristics are summarized in Table 2, with 17 of 18 studies published since 2011. Sixteen studies (89%)^15,16,17,18,19,20,21,22,23,24,25,26,27,28,29,31,32^ evaluated brain donation for research, 1 study (6%)^30^ focused on postmortem heart donation, and 1 study (6%)^31^ focused on all solid organs. A total of 12 124 participants across multiple self-reported races and ethnicities (eg, Black or African American, Chinese, Hispanic or Latiné, and White) were included. Fourteen studies (78%)^16,17,18,20,21,23,24,25,26,27,28,30,31,32^ reported data on Black or African American participants. Regarding study design, 17 articles (94%)^15,16,17,18,19,20,21,22,23,24,25,26,27,28,29,30,32^ used qualitative methods, including surveys (n = 7), interviews (n = 6), and focus groups (n = 6). Consent rates to organ donation were reported in 11 studies (61%).^17,18,20,21,22,23,24,25,28,29,30,32^

**Table 2.** Studies Evaluating the Perceptions, Beliefs, and Attitudes About Postmortem Organ Donation for Research

Source	Participants (No.)	Underrepresented group	Country	Study design	Organ	Consent rate	Study objectives	Themes
Bilbrey et al,^22^ 2018	Healthy participants (2557)	Hispanic or Latiné	US	Retrospective cohort study; qualitative mixed-methods study (focus group, survey, interview)	Brain	59% overall; 51% Hispanic or Latiné	Examine consent rate of brain donation among Hispanic or Latiné donors from an Alzheimer disease center; identify barriers and facilitators to brain donation among high-performing centers for Hispanic or Latiné brain donation	Mistrust; family involvement; information and misconceptions
Boise et al,^16^ 2017	Healthy participants (95); family of participants (34)	Black or African American; Chinese; Hispanic or Latiné; White	US	Qualitative study (focus group)	Brain	NA	Evaluate attitudes, beliefs, and experiences that affect receptivity to donating one’s brain for research across 4 racial or ethnic groups	Mistrust; family involvement; religious and cultural beliefs; information and misconceptions
Boise et al,^27^ 2017	Healthy participants (479)	Black or African American; Chinese; Hispanic or Latiné; White	US	Cross-sectional study; qualitative study (survey)	Brain	NA	Identify factors in willingness to assent to brain donation	Mistrust; religious and cultural beliefs; information and misconceptions
Caban-Holt et al,^26^ 2024	Healthy participants (227)	Black or African American	US	Cross-sectional study; qualitative study (survey)	Brain	NA	Investigate attitudes toward brain donation and perceptions of medical research that affect brain donation among African American individuals	Mistrust; family involvement; information and misconceptions
Darnell et al,^23^ 2011	Adults >65 y (46)	Black or African American	US	Qualitative study (structured interviews)	Brain	32% Black or African American	Assess knowledge of medical procedures, views on research, motives for participation, potential reasons for withdrawing from research, and willingness to donate their brain on death	Mistrust; information and misconceptions
Deep-Soboslay et al,^32^ 2019	Next of kin of deceased patients (1213)	Black or African American; White	US	Retrospective cohort study; qualitative study (survey)	Brain	70% overall; 57% Black or African American; 74% White	Determine factors associated with research participation of Black or African American families in postmortem human brain tissue donation for neuropsychiatric disorders as compared with White families	Information and misconceptions
Denninghoff,^30^ 2000	Family of decreased patients (14)	Black or African American	US	Cohort study; qualitative study (interview)	Heart	71% Black or African American	Enroll Black or African American patients who had sudden cardiac death to cardiac autopsy; understand barriers and facilitators to next-of-kin consent	Mistrust
Gamboa and Julion,^28^ 2021	Adults aged >65 y (755)	Black or African American	US	Cohort study; qualitative study (descriptive)	Brain	46% Black or African American	Describe the MARS’s brain donation challenges, processes, and successful brain procurement from older Black or African American adults	Family involvement; religious and cultural beliefs; information and misconceptions
Glover et al,^24^ 2020	Living adult organ donors (22)	Black or African American; Hispanic or Latiné; White	US	Qualitative study (focus group)	Brain	NA	Examine perceived impediments to completed brain autopsies among diverse older adults who have agreed to brain donation for clinical research	Family involvement; information and misconceptions
Jefferson et al,^25^ 2011	Adults aged >65 y (233)	Black or African American; White	US	Cross-sectional study; qualitative study (survey, scale)	Brain	49% Black or African American; 75% White	Examine factors associated with brain donation program participation among older Black or African American and White participants	Mistrust; religious and cultural beliefs; information and misconceptions
Jefferson et al,^21^ 2013	Adults aged >65 y (63)	Black or African American	US	Qualitative mixed-methods study (focus group, survey)	Brain	47% Black or African American; 75% White	Implement an informational protocol for older Black or African American participants and their family members about the benefits of clinical research and brain donation program participation in Alzheimer disease; quantitatively assess changes in knowledge, attitudes, and trust after implementation of protocol	Altruism; information and misconceptions
Lambe et al,^20^ 2011	Adults aged >55 y (15)	Black or African American	US	Qualitative study (focus group)	Brain	20%	Assess Black or African American adults’ knowledge and perceptions of brain donation; identify factors that relate to participating or not participating in a brain donation research program	Mistrust; altruism; family involvement; religious and cultural beliefs; information and misconceptions
Lentine et al,^31^ 2021	Family of deceased organ donors (690)	Black or African American; Hispanic or Latiné; White	US	Retrospective medical records review	All solid organs	NA	Examine donor factors associated with research authorization decline	Family involvement
Marrie et al,^29^ 2023	Adults with multiple sclerosis (4520)	White; other race and ethnicity	US; Canada	Cross-sectional study; qualitative study (survey)	Brain	62%	Evaluate attitudes about brain donation, reasons for participating or not participating in brain donation, and related communication preferences	Altruism; family involvement; information and misconceptions
Montoya et al,^15^ 2021	Healthy adults (15)	Hispanic or Latiné	US	Qualitative study (focus group)	Brain	NA	Examine the attitudes and beliefs about brain donation among adult children of older Hispanic or Latiné patients	Altruism; family involvement; religious and cultural beliefs; information and misconceptions
Morlett Paredes et al,^19^ 2023	Healthy adults (40)	Hispanic or Latiné	US	Qualitative study (interview)	Brain	NA	Assess Hispanic or Latiné adult attitudes about brain donation to inform methods by which researchers can increase consent	Altruism; religious and cultural beliefs; information and misconceptions
Schnieders et al,^18^ 2013	Adults (91)	Black or African American	US	Qualitative study (interview)	Brain	49% Black or African American	Collect information attitudes about research and brain donation among Black or African American patients	Mistrust; altruism; religious and cultural beliefs; information and misconceptions
Singh et al,^17^ 2022	Community-dwelling adults (1015)	Black or African	Ghana and Nigeria	Qualitative (interview)	Brain	19% Black or African	Ascertain the awareness, perspectives, and factors regarding sharing and informed consent preferences among community members in Ghana and Nigeria	Altruism; religious and cultural beliefs; information and misconceptions

Abbreviations: MARS, Minority Aging Research Study; NA, not applicable.

### Themes 

Thematic analysis of the 18 studies revealed 5 themes that informed participants’ decision to donate: information and misconceptions, mistrust, family involvement, religious and cultural beliefs, and altruism. We summarized the key findings by theme and how they served as barriers and facilitators to postmortem organ donation for research. Strategies to overcome barriers and leverage facilitators are highlighted in Table 3.

**Table 3.** Barriers and Facilitators to Postmortem Organ Donation

Theme	Major findings	Strategies
Information and misconceptions about the organ donation process	Lack of details on the process of organ donation Misconceptions about the process of tissue and organ procurement Limited understanding about the applications of the donated organ, particularly among those without disease	Provide longitudinal education with multiple touchpoints Develop culturally relevant materials Provide recruitment material in multiple languages across varying health literacies Inform participants with clear metrics of process and outcomes
Mistrust of the research and medical communities	Historical and intentional exclusion and mistreatment of the group are major barriers to organ donation Concerns that researchers may mishandle or mistreat the body after organ donation	Establish community-based outreach and engagement in developing study protocols Acknowledge and discuss historical injustices and medical racism Foster a diverse study team with representation from the community Clarify the informed consent process, and emphasize the ability to withdraw consent in research participation
Family involvement	Concerns that wishes of potential donors may not be ultimately granted by family Desire from both participants and family to understand the donor’s wishes about organ donation prior to consent	Develop family-centered information sessions Involve next of kin and family in information sessions, decision-making, and consent process Provide option of return of data to family
Religious and cultural beliefs	Concerns that donation would conflict with burial Desire for the body to be kept intact Concerns that organ donation would conflict with their own or their family members’ religious beliefs	Encourage clerical counsel and involvement Address the effect of donation on burial and religious rituals
Altruism	Belief that benefits to family and larger community are important to decision to donate Motivation that donation would benefit future generations and medical research would usher new cures and therapies	Highlight benefits of research to participants’ immediate community Discuss the implications of research publication for future generations Provide information to families on the key discoveries made possible through organ donation program

#### Information and Misconceptions About the Organ Donation Process

Sixteen studies (89%)^15,16,17,18,19,20,21,22,23,24,25,26,27,28,29,32^ evaluated how misconceptions and information delivery shaped attitudes toward organ donation for research. Many participants expressed informational paucity regarding the process of brain donation and misconceptions about the process, including how tissues are collected and used.^15,16,17,18,19,20,21,22,23,24,25,26^ Several studies reported lack of participant understanding of how healthy or diseased tissue could be of use to researchers.^16,20,22,24^ Most participants indicated that more information, such as knowing the step-by-step process, facilitated their willingness to donate.^15,16,18,20,21,24,27^

Many studies highlighted the importance of a culturally relevant, educational approach to donation decisions, including racially and ethnically diverse staff (mirroring the participant demographics), open and transparent discussion, and meeting participants in their communities or homes.^15,19,20,22,28^ A single semistructured focus group of the brain donation process among Black or African American individuals and next of kin encouraged discussion, but it did not increase knowledge, improve attitudes toward research, or elevate trust in medical researchers.^21^

In contrast, longitudinal approaches exemplified in the Minority Aging Research Study increased participation using a comprehensive strategy. The strategy consisted of educational sessions conducted by study personnel, individual sessions with each participant, and separate family meetings in person or by conference call.^22,28^ One study of patients with multiple sclerosis found that 75% of participants indicated a preference for receiving brain donation information from physicians.^29^

#### Mistrust of the Research and Medical Communities

Nine studies (50%)^16,18,20,22,23,25,26,27,30^ evaluated how trust shaped attitudes toward postmortem organ donation for research. Mistrust in the medical system was the leading reason for unwillingness to donate organs to researchers.^16,17,18,22,23,27^ Focus groups with Hispanic or Latiné community health workers revealed poor trust in researchers and fears that patients’ bodies would be mishandled, a concern shared by individuals across multiple races in another study.^22,27^ The mistreatment of historically marginalized communities contributed to reservations regarding organ donation for research among Black or African American and Hispanic or Latiné participants in several studies.^16,18,20,23,27^ Black or African American participants voiced concern that signing a legally binding contract (eg, consent to brain donation and autopsy) might restrict end-of-life health care choices or place agency in medical personnel whom they did not trust.^23^

Trust was also a facilitator of postmortem organ donation. In a small study of Black or African American patients who had sudden cardiac death, a high rate of consent (71%) by family was partially attributed to strong community relationships between the participants and staff who reflected the people in the community.^30^ One survey of Black or African American community members found high levels of trust in research and researchers, which was attributed to extensive community outreach and established relationships with participants.^26^ Most studies with well-trained staff, grounded in building rapport, were most effective at reducing power differential dynamics and promoting trust.^21,22,23,28^

#### Family Involvement

Nine studies (50%)^15,16,20,22,24,26,28,29,31^ highlighted that family involvement in the recruitment and consent processes served as both a barrier and a facilitator. In focus groups, family members acknowledged that discussing brain donation was difficult and they were concerned that the donor’s wishes would not be granted.^16^ In a study of older Black or African American and Hispanic or Latiné individuals, participants were concerned their families would not implement their brain donation plan due to family desires for traditional interments. Participants expressed that families may perceive brain donation as inconvenient during the grieving process.^24^ Older Black or African American donors were also hesitant to discuss organ donation with next of kin due to fear of burdening their family and fear of resistance.^20^ In a retrospective study of 690 deceased donors, research authorization by family members was declined in 11% of all brain donations, with rates of decline noted at 9% for White donors, 16% for Black or African American donors, and 24% for donors from other racial minority groups.^31^ Research authorization decline was highest among donors older than 65 years and when donors themselves did not provide first-person authorization.^31^

In an cohort of older Black or African American participants who consented to brain donation, family concerns contributed to the greatest source of delays.^28^ Reasons for delay included family members being uninformed of the donor’s donation wish, having multiple designated contact persons, or contact persons holding up the procurement until further information or religious permission was granted.^28^ In a survey of patients with multiple sclerosis, numerous demographic factors, including higher educational level and higher annual household income, were associated with willingness to involve family in the decision-making process.^29^

Family involvement was also a facilitator of postmortem organ donation.^15,16,22,26^ Next of kin indicated that knowing the preference of potential donors facilitated support of their decisions and reduced guilt.^16^ Studies of Hispanic or Latiné participants revealed that family involvement in research recruitment, informational sessions, and the consent process were essential to engaging and enrolling Hispanic or Latiné participants in research brain donation programs.^15,22^

#### Religious and Cultural Beliefs

Nine studies (50%)^15,16,17,18,19,20,25,27,28^ discussed how religious and cultural beliefs served as barriers in brain donation for research. Organ donation was viewed to conflict with participants’ religious customs, particularly the preservation of the body or keeping it intact without alteration and appearance of the body at the time of final rites.^15,16,17,18,19,20,25,27,28^ Individuals also expressed hesitation on whether removal of the brain would affect the soul or metaphysical ascent to the afterlife.^16,17,19,20^ Compared with White participants, Black or African American participants were more likely to believe that “most religions do not support brain donation”; however, among White respondents, a higher level of intrinsic religiousness was an unfavorable factor in brain donation status.^25^ Two studies noted that older-generation Hispanic or Latiné participants viewed religious beliefs as a potential barrier to brain donation.^15,19^ In contrast, in 1 study, Hispanic or Latiné participants expressed a higher willingness for brain donation compared with White and Black or African American participants, suggesting that barriers related to religious beliefs may be overcome.^27^ Individuals with religion-based hesitations voiced openness to brain donation after receiving information that donation would have limited consequences for body appearance and burial.^18,20^

#### Altruism

Altruism was a significant facilitator of organ donation in 7 studies (39%).^15,17,18,19,21,25,29^ In interviews, older Black or African American participants cited a benefit to themselves or family members (53%) and to others (21%) as a motivator to organ donation for research.^18^ Two additional studies of older Black or African American participants found that the primary incentive for brain donation was the possibility of family benefit, specifically in families with a history of Alzheimer disease.^20,21^ In focus groups of Hispanic or Latiné adults, inclusion of the benefits of brain donation to families and future generations was perceived as facilitating brain donation.^15^ In interviews with Hispanic or Latiné family members, 57% of participants expressed a greater willingness to donate knowing it could help researchers find a cure for Alzheimer disease from which their family, future generations, and humanity could benefit.^19^

## Discussion

This systematic review identified 5 major themes across studies that played a role in the attitudes and beliefs about postmortem organ donation for research among adults from historically marginalized races and ethnicities. We observed that personal beliefs, such as religious customs, and mistrust of the medical system were barriers that could be minimized. Misconceptions about the process and purpose of organ donation for research were common barriers. Providing facts regarding the organ donation procedures, emphasizing the importance of postmortem organ donation for advancing science, and providing culturally relevant information on organ donation longitudinally could overcome concerns. Family involvement served as both a barrier and a facilitator. Participants expressed fears of discussing organ donation with family and concerns that their wishes may not be honored. Unengaged family members may not value the participant’s donation wishes and advanced directive. Therefore, most studies suggest early family engagement to aid in decision-making and promote consent. Altrustic perception and understanding that postmortem organ donation for research would benefit the participant’s family, future generations, and community were a significant facilitator to consent.

The barriers identified in this review, including mistrust, religious beliefs, and misconceptions about organ donation for research, are consistent with parallel literature examining attitudes about organ donation for transplant.^10,33,34,35,36^ Attitudes and beliefs about organ donation represent both a challenge and an opportunity for scientists to increase engagement with disinvested groups. Several strategies for overcoming barriers have been noted in this review and from prior literature on organ transplant programs. For example, longitudinal programs that emphasize mutual trust and transparency in the process of recruitment can combat mistrust from marginalized groups in the medical and research communities.^20,37^ Culturally sensitive promotion of organ donation for research, including acknowledgment of the historical mistreatment of research participants and the potential benefits to their community, is essential when discussing organ donation.^21,37,38^ Other studies note that repeated engagement with the community, as well as racial and ethnic representation among research staff, can reduce feelings of a power differential and promote trust.^15,19,20,21,22,28^ Furthermore, leveraging the use of peers in recruitment, including local clergy and religious leaders, has been shown to increase interest and participation of individuals from diverse backgrounds in enrollment in organ donation programs.^39,40,41^

The findings also highlighted the importance of education and information in the process of organ donation recruitment. Most participants expressed that having detailed information about donation, including the step-by-step process, how samples might be used, and the benefits to the larger community, facilitated their willingness to donate.^15,16,18,20,21,24,27^ Moreover, individuals with religious and cultural hesitations about donation often changed their opinion after discussion about the limited implications of brain donation for burial.^18,20^ Prior studies in organ donation for transplant have demonstrated that culturally relevant messaging delivered through video is particularly effective in promoting rates of consent.^42,43^ Recruitment materials in the languages spoken within the community are also needed to increase recruitment of individuals for whom English is not their first or preferred language.^22^

Family involvement is particularly relevant to postmortem organ donation given that the next of kin must provide consent at the time of death. In the analysis, individuals who viewed family as a barrier expressed concern that their wishes may not be honored or may place undue burden on their family during a vulnerable time.^16,20,24^ Both older Black or African American and Hispanic or Latiné participants often felt that discussing brain donation with their families was an important part of their decision-making process.^16,22,44^ When families were engaged early in the process and participants’ wishes were well known to their family, participants were more likely to consider donation.^15,22^ Although individual consent is the cornerstone of organ donation ethics, studies show that the majority of the public supports family involvement in organ donation.^45^

The available literature suggests a need to increase the number and quality of studies evaluating barriers and facilitators to all organ donation for biobanking, not just brain donation. While it may be reasonable that strategies to foster brain donation may be transferrable to other organs, such approaches need to be tested empirically, and organ-specific adaptations need to be evaluated. As the future of genomics and biobanking moves forward, postmortem tissue procurement from a representative sample of the general population will be essential.

### Strengths and Limitations

This study has several strengths. The search strategy included medical librarian–guided strict eligibility criteria specific to organ donation for research and included a large sample of participants from historically marginalized racial and ethnic groups. Based on PRISMA guidelines, we used a standardized approach with multiple independent reviewers to screen and review articles for thematic analysis, although we acknowledge the subjective nature of the analysis.

The study also has limitations. The available evidence presented limitations in study quality regarding generalizability, reliability, validity, and data analysis. Because of the heterogeneity of study designs and qualitative information, we did not use formal meta-analytic techniques. Additionally, most participants were possible donors for brain donation. Individuals may have different perceptions for the brain than other solid organs, particularly since the brain is often perceived or believed to be the source of personhood. While Black or African American and Hispanic or Latiné individuals were well represented in the studies, other racial and ethnic minorities, such as American Indian or Alaska Native, Asian, and Native Hawaiian or Other Pacific Islander, were less or not at all represented, thus limiting the generalizability of results. Nearly all of the studies were conducted solely in the US (Table 2), with only 1 study^17^ conducted in Ghana and Nigeria, limiting the global perspective and examination of how beliefs of structurally marginalized communities differ across countries. Generalizations across racial and ethnic identity groups diminish the importance of intersectionality with other identities and demographic characteristics, such as sex, gender, socioeconomic status, immigration status, language, religion, and educational level. Data are lacking on intersectionality in organ donation for research.

## Conclusions

This systematic review found barriers to organ donation for research, including mistrust, religious customs, organ procurement, and burial considerations, in individuals from historically marginalized racial and ethnic groups. However, agency and information about the donation process and engagement of the participants and next of kin in decision-making may facilitate greater organ donation in the research community and in longitudinal biobank programs. Leveraging and sharing information that addresses barriers and facilitators to donor recruitment practices may yield more effective and ethical strategies to increase the diversity of translational research participation.

## Notes

Supplement 1. **eFigure**. Translational Research Continuum with Postmortem Studies
eReferences

Supplement 2.
Data Sharing Statement
