# Supplementary material for: Organ Donation for Research Biobanking Among Historically Marginalized Racial and Ethnic Groups: A Systematic Review
Source: JAMA Netw Open. 2025 May 27;8(5):e2512133. doi: 10.1001/jamanetworkopen.2025.12133 (PMC12117466; doi:10.1001/jamanetworkopen.2025.12133)
Supplement: Supplement 1. — eFigure. Translational Research Continuum with Postmortem Studies eReferences [file jamanetwopen-e2512133-s001.pdf]

Supplemental Online Content

Toro C, Eromosele OB, Flynn DB, et al. Organ donation for research biobanking among historically marginalized racial and ethnic groups: a systematic review. *JAMA Netw Open*. 2025;8(5):e2512133. doi:10.1001/jamanetworkopen.2025.12133

eFigure.Translational Research Continuum with Postmortem Studies

eReferences

This supplemental material has been provided by the authors to give readers additional information about their work.

© 2025 Toro C et al. *JAMA Network Open*

eFigure.Translational Research Continuum with Postmortem Studies

Translational research is informed by basic science discoveries, including those using postmortem tissues, in preclinical studies.[1,2] The translational research continuum spans four phases with individuals from historically and intentionally excluded groups being underrepresented across all aspects of the continuum.[3-8]

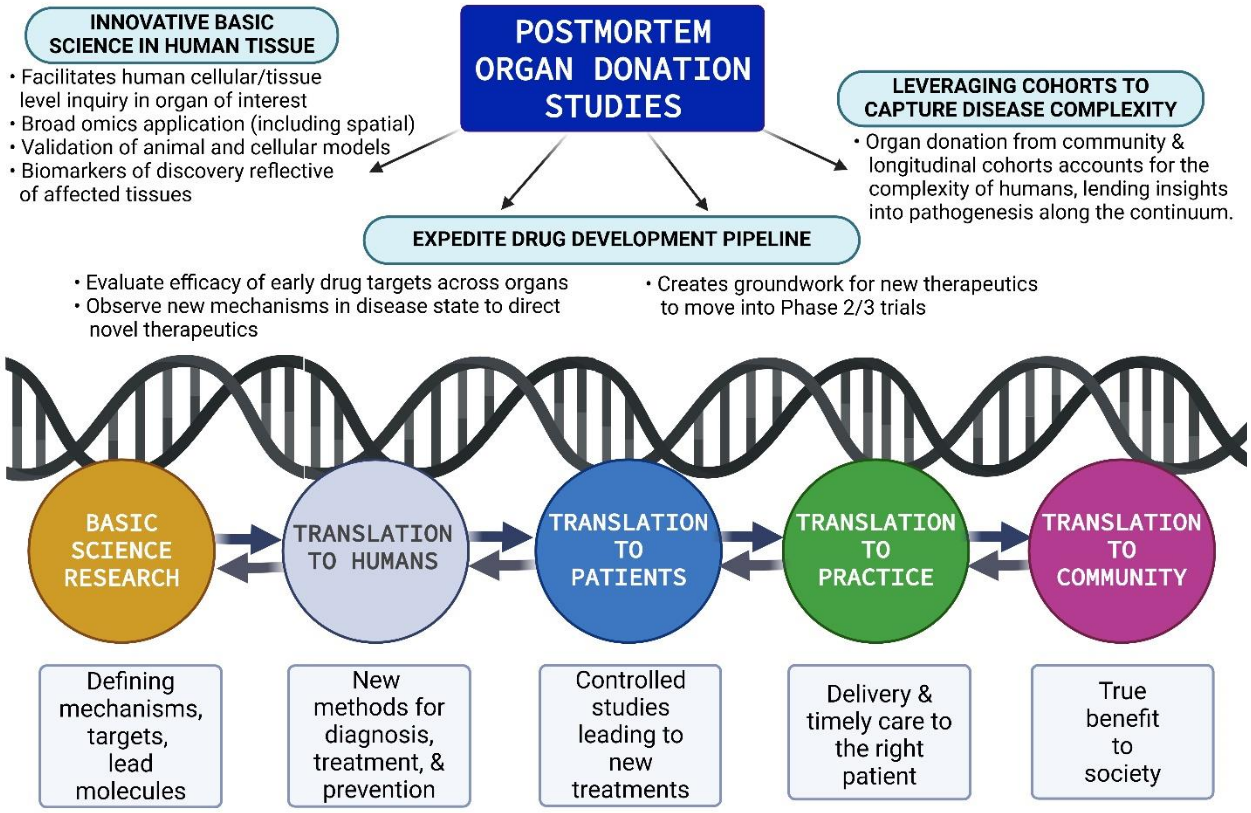

© 2025 Toro C et al. *JAMA Network Open*

eReferences

1. Khoury MJ, Gwinn M, Yoon PW, Dowling N, Moore CA, Bradley L. The continuum of translation research in genomic medicine: how can we accelerate the appropriate integration of human genome discoveries into health care and disease prevention? *Genet Med*. 2007;9:665-674. doi: 10.1097/GIM.0b013e31815699d0
2. Drolet BC, Lorenzi NM. Translational research: understanding the continuum from bench to bedside. *Transl Res*. 2011;157:1-5. doi: 10.1016/j.trsl.2010.10.002
3. Chen MS, Jr., Lara PN, Dang JH, Paterniti DA, Kelly K. Twenty years post-NIH Revitalization Act: enhancing minority participation in clinical trials (EMPaCT): laying the groundwork for improving minority clinical trial accrual: renewing the case for enhancing minority participation in cancer clinical trials. *Cancer*. 2014;120 Suppl 7:1091-1096. doi: 10.1002/cncr.28575
4. Burchard EG, Oh SS, Foreman MG, Celedon JC. Moving toward true inclusion of racial/ethnic minorities in federally funded studies. A key step for achieving respiratory health equality in the United States. *Am J Respir Crit Care Med*. 2015;191:514-521. doi: 10.1164/rccm.201410-1944PP
5. Murthy VH, Krumholz HM, Gross CP. Participation in cancer clinical trials: race-, sex-, and age-based disparities. *JAMA*. 2004;291:2720-2726. doi: 10.1001/jama.291.22.2720
6. Sardar MR, Badri M, Prince CT, Seltzer J, Kowey PR. Underrepresentation of women, elderly patients, and racial minorities in the randomized trials used for cardiovascular guidelines. *JAMA Intern Med*. 2014;174:1868-1870. doi: 10.1001/jamainternmed.2014.4758
7. Fatumo S, Chikowore T, Choudhury A, Ayub M, Martin AR, Kuchenbaecker K. A roadmap to increase diversity in genomic studies. *Nat Med*. 2022;28:243-250. doi: 10.1038/s41591-021-01672-4
8. Sirugo G, Williams SM, Tishkoff SA. The Missing Diversity in Human Genetic Studies. *Cell*. 2019;177:26-31. doi: 10.1016/j.cell.2019.02.048

© 2025 Toro C et al. *JAMA Network Open*
