# Supplementary material for: Organ Donation for Research Biobanking Among Historically Marginalized Racial and Ethnic Groups: A Systematic Review
Source: JAMA Netw Open. 2025 May 27;8(5):e2512133. doi: 10.1001/jamanetworkopen.2025.12133 (PMC12117466; doi:10.1001/jamanetworkopen.2025.12133)
Supplement: Supplement 2. — Data Sharing Statement [file jamanetwopen-e2512133-s002.pdf]

Data Sharing Statement

Toro. Organ Donation for Research Biobanking Among Historically Marginalized Racial and Ethnic Groups. *JAMA Netw Open*. Published May 27, 2025.
doi:10.1001/jamanetworkopen.2025.12133

Data
Data available: No

Additional Information
Explanation for why data not available: Not applicable
